# Identification Method for Seated Balance Control Based on Horizontal Support-Surface Sway and Dual Force-Platform Measurements

**DOI:** 10.3390/s26196007

**Published:** 2026-09-22

**Authors:** Mohamed Elsaroukh, Motomichi Sonobe, Tsukasa Okaji

**Affiliations:** 1 Department of Intelligent Mechanical Systems Engineering, Kochi University of Technology, 185 Miyanokuchi, Tosayamada, Kami, Kochi 782-8502, Japan; mohamedelsaroukh@sh-eng.menofia.edu.eg (M.E.);; 2 Faculty of Engineering, Menoufia University, Shebin El-Kom 32511, Egypt

**Keywords:** seated posture, center of mass, force platform, balance identification, head acceleration feedback, time delay

## Abstract

Identifying seated balance control may contribute to estimation of time delay and evaluation of vestibular weighting. However, few practical methods exist for measuring seated body motion or identifying seated balance control. This study aimed to estimate upper-body center of mass (COM) displacement during sitting under horizontal sway of the support surface using a practical measurement method and to identify a seated balance control system for each individual. Two force platforms under the buttocks and feet were synchronized with a support surface accelerometer. Under unpredictable horizontal perturbations composed of a multi-cosine waveform below 1.50 Hz, balance control was identified in an eyes-closed condition using a parametric model incorporating delayed COM state feedback and horizontal head acceleration feedback, the latter representing otolith-mediated detection of horizontal head acceleration. When validated against an optical motion capture system (five participants), the identified parameters exhibited an average relative error of less than 5%. A two-day reproducibility test (25 participants) yielded intraclass correlation coefficients (ICC(1,1)) of the four identified parameters ranging from 0.57 to 0.73, indicating moderate reliability. The identified head acceleration feedback gain may indicate head acceleration sensitivity, even among healthy adults.

## 1. Introduction

Identifying individual balance control systems in seated postures [1] may contribute to estimation of time delay and evaluation of vestibular weighting [2,3,4,5]. A key requirement for identification is the measurement of the upper-body center of mass (COM) under external perturbations. Conventional approaches estimate COM dynamics from segmental kinematics using optical motion capture systems [6,7]. However, these optical systems are costly, space-consuming, suffer from marker errors, and are often impractical for routine clinical use or when experiments involve moving platforms [8,9]. Therefore, an accessible sensor-based measurement technique that can capture upper-body dynamics is required.

To overcome these measurement issues, indirect estimation using force platforms is a practical alternative method. Our previous study proposed a method for estimating COM displacement during standing using measurements obtained from a combination of force platforms and inertial sensors [10]. Furthermore, we extended this method to propose a technique for estimating upper-body COM displacement using dual force platforms [11]. Importantly, these methods can also be applied when the support surface moves horizontally. This measurement approach can be used to identify individual balance control systems based on general system identification techniques [12,13,14].

To identify a system with a time delay, periodic perturbations should be applied, and a balance control law should be assumed [15,16,17,18,19]. To identify an individual balance control system, it is necessary to simplify the system by reducing its degrees of freedom and control objectives [20,21]. For the standing posture, our previous study proposed an identification method that considered only COM control by limiting the frequency band below 0.7 Hz to apply a single inverted pendulum model [22]. However, this approach has two major limitations: first, the narrow frequency range makes it difficult to estimate the time delay; second, it cannot capture individual differences in head acceleration control. In contrast, identifying a balance control model for sitting can utilize a broader frequency range of up to approximately 2 Hz because the single inverted pendulum assumption remains valid in this range. Furthermore, the greater postural stability of the sitting posture allows for the application of larger horizontal accelerations, thereby making head acceleration control more critical [23,24,25]. Because the COM control is expressed in a moving coordinate system and the head acceleration control is expressed in a stationary coordinate system [26], it is theoretically possible to distinguish between the two objectives.

This study proposes a practical measurement and identification technique for seated balance control based on dual force platform measurements and the constrained dynamic structure of the seated posture. Using unpredictable horizontal perturbations in the sagittal plane, the upper-body COM motion was indirectly estimated from the force and support surface acceleration data. Subsequently, frequency-domain analysis was applied to an inverted pendulum model to identify the COM feedback gain, neuromuscular time delay, and horizontal head acceleration feedback gain [27,28,29]. Measurement validity was confirmed against simultaneous optical motion capture, and the reproducibility of the identified parameters was evaluated across two days. Finally, to investigate the characteristics of the identified model, we examined the correlation between COM control and head acceleration control and performed a sensitivity analysis of the identified model.

## 2. Methods

### 2.1. Participants

Twenty-five healthy males (age: 22.8 ± 1.1 years; height: 173.8 ± 7.0 cm; body mass: 65.9 ± 10.5 kg) voluntarily participated in this study. For reproducibility evaluation, all participants (Subject 1–25) completed ten trials per day across two non-consecutive days. Among them, five participants (Subject 21–25) also underwent simultaneous optical motion capture measurements on the first day for comparative validation. The inclusion criteria for the participants were the absence of any known neurological, musculoskeletal, or vestibular disorders. Written informed consent was obtained from all participants. This study was approved by the Ethics Committee of Kochi University of Technology (Approval No. 239). All experimental procedures were performed in strict accordance with the ethical principles outlined in the Declaration of Helsinki and adhered to institutional guidelines for human research.

### 2.2. Experimental Setup

A custom-designed electric cart delivered precise horizontal perturbations in the sagittal plane to evaluate seated postural control dynamics (Figure 1). The cart motion was driven by a 400 W brushless DC motor (EC60, Maxon Motor, Sachseln, Switzerland) controlled using a control unit (EPOS2 70/10, Maxon Motor). The seat of the cart was elevated to 450 mm above the foot-support surface. To characterize the dynamic response of the seated postural control system, perturbations were applied to the cart using a multi-frequency sway protocol. The cart acceleration *Ẍ_s_* was generated as a superposition of 30 discrete cosine waves with frequencies *f_k_* ranging from 0.05 to 1.50 Hz in increments of 0.05 Hz, as follows:

(1) $${\ddot{X}}_{s}(t)=A\sum_{k=1}^{30}\cos(\omega_{k}t+\phi_{k}),{\;\;\;\;\;\;\;\;\omega }_{k}=2\pi f_{k}$$

where *ω_k_* denotes the angular frequency of the *k*th component. Amplitude *A* was set to a constant value across all frequencies such that the resulting maximum acceleration was approximately 0.3 m/s^2^. Phase *ϕ_k_* was determined to make the resulting acceleration signal approximate a Gaussian amplitude distribution (SD = 0.15 m/s^2^). Five distinct phase configuration sets were employed across the experimental trials to enhance stimulus unpredictability. Each experimental trial lasted 65 s.

To quantify the postural responses to the applied perturbations, the experimental cart was instrumented with two force platforms (TF-3040, Tec Gihan, Kyoto, Japan) positioned beneath the buttocks and feet of the participants. The horizontal acceleration and position of the cart were continuously recorded using a high-precision triaxial accelerometer (ADXL355, Analog Devices, Wilmington, MA, USA) and a rotary encoder (HEDL 9140, Maxon Motor), respectively. For comparative validation of the estimated COM, an optical motion capture system (Qualisys, Gothenburg, Sweden) with eight Miqus cameras was used simultaneously; forty-one reflective markers were placed on the participant’s body, and four additional markers were attached to the cart. All measurements from these sensors and the optical system were synchronized and sampled at a frequency of 100 Hz.

The participants were instructed to sit with their knees flexed at approximately 90° and their arms resting naturally at their sides. The participants performed the task with their eyes closed while wearing noise-canceling headphones (WH-1000XM4, Sony, Tokyo, Japan) to mask the cart motor noise. The eyes-closed condition was adopted to eliminate large individual variations in visual feedback [30,31,32], which prevents the identification of balance systems for all participants under the assumption of a single control law. Additionally, participants wore noise-canceling headphones to prevent them from using motor sounds as auditory cues to anticipate cart movement, which could otherwise lead to control behaviors similar to those observed with visual feedback. During each daily session of ten trials, a rest period of at least 5 min (extended upon request) was provided after each set of five trials.

### 2.3. COM Estimation Method

The seated posture in the sagittal plane was modeled as a multi-link system, with the upper-body represented as a single-link inverted pendulum pivoting at the waist (near the fifth lumbar vertebra, L5) as shown in Figure 2. The model comprises the upper-body (including the trunk, head, and arms), pelvis, thighs, lower legs, and feet. We assumed that the upper-body moved independently, whereas all other body segments were rigidly fixed to the platform. The head was assumed to lie on the extension of the line connecting the waist and the upper-body COM. According to previous studies [23,24], the upper-body can be treated as a rigid body at frequencies of 2 Hz or lower. The motion is expressed in a stationary coordinate system (*O*–*XYZ*) and a moving coordinate system (*p*–*xyz*), where the origin *p* is fixed to the cart. Both frames were aligned such that the *X*/*x*-axes pointed forward, the *Y*/*y*-axes leftward, and the *Z*/*z*-axes were upward.

The measurement system recorded the three-axis reaction forces (*R_x_*_1_, *R_y_*_1_, *R_z_*_1_; *R_x_*_2_, *R_y_*_2_, *R_z_*_2_) and moments of force (*N_x_*_1_, *N_y_*_1_, *N_z_*_1_; *N_x_*_2_, *N_y_*_2_, *N_z_*_2_) under the pelvis (subscript 1) and feet (subscript 2), as well as the cart acceleration ${\ddot{X}}_{s}$. From the equations of motion, the COM acceleration and displacement of the upper-body in moving coordinates are obtained as follows:

(2) $${\ddot{\tilde{x}}}_{u}(t)=-\frac{1}{m_{u}}\left\{M{\ddot{X}}_{s}(t)+R_{x1}(t)+R_{x2}(t)\right\}$$

(3) $$\begin{array}{c} {\tilde{x}}_{u}\left(t\right)=-\frac{1}{m_{u}g}\left\{K_{1}{\ddot{\tilde{x}}}_{u}\left(t\right)+K_{2}{\ddot{X}}_{s}\left(t\right)+K_{3}-N_{y1}-N_{y2}\right.\\ \;\;\;\;\;\;\;\;\;\;\;\;\;\;\;\;\;\;\;\;\;\;\;\;\;\;\;\;\;\;\;\;\;\;\;\;\;\left.+\left(l_{p}+l_{f}-L_{f}-L_{l}\right)R_{x1}(t)+l_{f}R_{x2}(t)-L_{t}R_{z1}(t)\right\} \end{array}$$

(4) $$\left.\begin{array}{c} K_{1}=-\frac{J_{u}}{l_{u}}+m_{u}\left(l_{p}+l_{f}-l_{u}-L_{p}-L_{l}-L_{f}\right)\\ K_{2}=m_{u}\left(l_{p}-l_{u}-L_{p}\right)+M\left(l_{f}-L_{l}-L_{f}\right)+m_{f}\left(L_{l}+L_{f}-l_{f}\right)+m_{l}\left(L_{l}-l_{l}\right)\\ K_{3}=\left(-m_{u}L_{t}-m_{p}L_{t}-m_{t}l_{t}\right)g \end{array}\right\}$$

where ${\tilde{x}}_{u}$ is the estimated upper-body COM displacement. For each segment, including the upper-body (subscript *u*), pelvis (*p*), thigh (*t*), lower leg (*l*), and foot (*f*), the segment mass is denoted by *m*, segment length by *L*, and COM height by *l*. *J_u_* denotes the moment of inertia around the upper-body COM. These physical parameters were derived as functions of the subject’s height *H* [m] and body mass *M* [kg], as presented in Table 1 [33,34]. This method enables a high-accuracy estimation of the upper-body COM displacement without using an optical motion capture system [11].

### 2.4. Parametric Model

This study identified a balance control system based on a parametric model to integrate mechanical dynamics with a neurophysiological control system. The mechanical model is regarded as a single-link inverted pendulum. The equation of motion is given by

(5) $$\left(\frac{J_{u}}{l_{u}}+m_{u}l_{u}\right){\ddot{x}}_{u}(t)-m_{u}gx_{u}(t)=-m_{u}l_{u}{\ddot{X}}_{s}(t)+N_{w}(t)$$

The waist torque *N_w_* is hypothesized as follows:

(6) $$N_{w}\left(t\right)=-k_{p}x_{u}\left(t-t_{d}\right)-k_{d}{\dot{x}}_{u}\left(t-t_{d}\right)-k_{h}\frac{L_{u}}{l_{u}}{\ddot{x}}_{u}\left(t-t_{d}\right)-k_{h}{\ddot{X}}_{s}\left(t-t_{d}\right)$$

where *k_p_* is the proportional gain, *k_d_* is the derivative gain, *k_h_* is the head acceleration feedback gain, and *t_d_* is the time delay. The first and second terms on the right-hand side in (6) denote the delayed state feedback control of the COM. In contrast, the third and fourth terms correspond to the delayed feedback of the head acceleration in the stationary coordinate system. Because these control objectives conflict during the support surface sway, their gains can be identified independently.

From (5) and (6), a transfer function whose input is the platform acceleration and output is the upper-body COM velocity, is derived as follows:

(7) $$G\left(s\right)=\frac{-\left(k_{h}e^{-st_{d}}+m_{u}l_{u}\right)s}{\left(\frac{J_{u}}{l_{u}}+m_{u}l_{u}+k_{h}\frac{L_{u}}{l_{u}}e^{-st_{d}}\right)s^{2}+k_{d}e^{-st_{d}}s+k_{p}e^{-st_{d}}-m_{u}g}$$

The corresponding block diagram is shown in Figure 3. This formulation is well suited for fitting the model to the experimental frequency response function (FRF). The unknown individual parameters *k_p_*, *k_d_*, *k_h_*, and *t_d_* were identified using the transfer function *G*(*s*). While individual parameter identification methods for standing posture based on a single inverted pendulum model typically involve five unknown parameters [21,22], the proposed seated model reduces the number of unknowns to four, which is expected to improve the practical identifiability and consistency of the parameter estimation. Since the values of these feedback gains depend on the height and body mass of an individual, they were normalized as follows:

(8) $$K_{p}=\frac{k_{p}}{\frac{J_{u}}{l_{u}}+m_{u}l_{u}},K_{d}=\frac{k_{d}}{\frac{J_{u}}{l_{u}}+m_{u}l_{u}},K_{h}=\frac{k_{h}L_{u}}{J_{u}+m_{u}l_{u}^{2}}$$

where *K_p_*, *K_d_*, and *K_h_* represent the normalized control parameters corresponding to *k_p_*, *k_d_*, and *k_h_*, respectively.

### 2.5. Data Processing

The raw signals of the horizontal reaction forces and platform acceleration were first preprocessed to eliminate baseline offsets. The measured horizontal reaction forces (*R_x_*_1_ and *R_x_*_2_) were corrected by subtracting the inertial forces of the measuring plates, which were estimated based on the measured platform acceleration.

Frequency analysis was conducted on 60 s of data after discarding the initial 5 s of the recorded data. To calculate the Fourier coefficients of the COM velocity, which were regarded as the output of the FRF, the COM displacement and acceleration obtained from (2) and (3) were utilized. Since the estimation accuracy is higher for COM displacement at low frequencies and for COM acceleration at high frequencies, the estimated values were combined using 0.65 Hz as the boundary.

The unknown control parameters were identified using bounded nonlinear least-squares optimization. The parameter estimation and analysis were restricted to 0.3–1.5 Hz. FRFs below 0.3 Hz were excluded from the analysis because the eyes-closed condition caused low-frequency COM fluctuations to become irregular, resulting in greater variability in the FRFs within this frequency range. In addition, to promote the stability of the control system, the normalized head acceleration feedback gain was constrained as 0 ≤ *K_h_* ≤ 1 [35].

### 2.6. Verification

Statistical analyses were performed to evaluate the reproducibility and validity of the proposed method. Reproducibility was assessed using the intraclass correlation coefficient (ICC(1, 1); one-way random-effects model, absolute agreement) calculated between the two sessions for the identified parameters. The ICC(1, 1) was specifically chosen to enable direct comparison with previous research on standing [22] and because it provides a more conservative evaluation of reliability than the ICC(3, 1) model. To assess validity, the presence of a trade-off relationship between the COM and head acceleration controls was examined. The characteristic equation of the system identified for each individual is derived from (7) as follows:

(9) $$\left(\frac{J_{u}}{l_{u}}+m_{u}l_{u}+k_{h}\frac{L_{u}}{l_{u}}e^{-\lambda t_{d}}\right)\lambda^{2}+k_{d}e^{-\lambda t_{d}}\lambda +k_{p}e^{-\lambda t_{d}}-m_{u}g=0$$

Defining *σ*_max_ as the maximum value of the real part of the characteristic root *λ*, a smaller *σ*_max_ (<0) indicates that the weight of COM control is larger. Therefore, the Pearson correlation coefficient between *σ*_max_ and *K_h_* was examined.

### 2.7. Sensitivity and Parameter Identifiability

The local parameter sensitivity and practical identifiability of the four-parameter postural control model ***q*** = [*k_p_*, *k_d_*, *k_h_*, *t_d_*]*^T^* were evaluated in the frequency domain [36]. The sensitivity analysis was performed over the selected identification bandwidth (0.3–1.5 Hz, in 0.05 Hz increments) to assess whether it contained sufficient information for reliable estimation of the four model parameters. The model transfer function *G*(*jω*) was analytically differentiated with respect to each model parameter using symbolic differentiation. The resulting derivatives were evaluated at the discrete angular frequencies *ω_k_* (*k* = 1, …, *N*) over the analyzed frequency range, yielding a complex sensitivity matrix ***S*** [37].

(10) $$S_{k,i}=\frac{\partial G({j\omega }_{k})}{{\partial q}_{i}}$$

where *N* is the number of analyzed frequency samples, and *q_i_* denotes the *i*-th element of the parameter vector ***q***. To enable a direct comparison among parameters with different physical units, the sensitivities were normalized as follows:

(11) $${\tilde{S}}_{k,i}=\frac{q_{i}}{G(j\omega_{k})}\;S_{k,i}$$

This provides a dimensionless measure of the relative sensitivity of the transfer function with respect to each parameter. The normalized sensitivity matrix is then defined as:

(12) $$\tilde{S}=\left[\begin{array}{cccc} {\tilde{S}}_{1}\left(\omega_{1}\right) & {\tilde{S}}_{2}\left(\omega_{1}\right) & \ldots & {\tilde{S}}_{4}\left(\omega_{1}\right)\\ {\tilde{S}}_{1}\left(\omega_{2}\right) & {\tilde{S}}_{2}\left(\omega_{2}\right) & \ldots & {\tilde{S}}_{4}\left(\omega_{2}\right)\\ \vdots & \vdots & \ddots & \vdots \\ {\tilde{S}}_{1}\left(\omega_{N}\right) & {\tilde{S}}_{2}\left(\omega_{N}\right) & \ldots & {\tilde{S}}_{4}\left(\omega_{N}\right) \end{array}\right]$$

where each row corresponds to a discrete frequency sample, and each column corresponds to a model parameter. Because the normalized sensitivity matrix is complex-valued, its real and imaginary parts are combined to form an augmented Jacobian matrix [38]. Singular value decomposition (SVD) was then applied to evaluate the practical identifiability. Specifically, the numerical rank of the Jacobian was used to determine the number of independently identifiable parameter combinations, whereas the condition number was used to assess the parameter conditioning and correlation. Higher condition numbers indicate poorer practical identifiability [39].

## 3. Results

### 3.1. Conformity of the Identified System

The proposed method successfully identified the seated balance control system for all participants on both test days under eyes-closed conditions. Figure 4 presents the FRF (input: platform acceleration, output: COM velocity) of both the experimental results and identified system for a representative participant on days 1 and 2. The mean and standard deviation of the experimental results across the ten daily trials are indicated by black circles and error bars, respectively, and the identified system responses are represented by the blue lines. By limiting the frequency range to above 0.3 Hz, the identified system closely matched the experimental results.

### 3.2. Validation of COM Estimation Method

The COM estimation method was validated through experimental trials involving five participants, each of whom performed ten trials. Figure 5 presents the FRF for a representative participant (Subject 21), comparing the results obtained from the proposed method with those obtained using the optical motion capture system. Across the analyzed frequency range, the two estimation approaches exhibited close agreement in both magnitude and phase. Notably, although Subject 21 demonstrated the largest estimation difference among the five participants (Subject 21–25), the FRF profiles still exhibited strong alignment, underscoring the reliability of the proposed method, even in the worst case.

To further quantify the method-level consistency, the identified control parameters were estimated using both approaches and compared in terms of the relative estimation errors. The mean and standard deviation of the relative errors across participants were as follows: *K_p_* = 4.01 ± 2.80%, *K_d_* = 2.83 ± 3.95%, *K_h_* = 2.09 ± 11.38%, and *t_d_* = 4.24 ± 7.02%. Although the standard deviation of the *K_h_* error exceeded 10%, this variability may be related to the difficulty in accurately measuring the upper-body COM motion at high frequencies using an optical motion capture system. The overall magnitude of the estimation errors remained low, demonstrating that the proposed COM estimation method provides sufficient and consistent accuracy for parameter identification without relying on optical motion capture data. This comparison should be interpreted as a method-level validation of the COM estimation approach, considering the limited number of participants involved.

### 3.3. Reproducibility of the Identified System

Figure 6 shows the parameters identified for all participants over the two experimental days. The normalized head acceleration feedback gain (*K_h_*), ranging from 0 to 1 by definition, was greater than 0.5 for the majority of participants; however, distinct individual differences were still observed even among healthy participants. Furthermore, the neuromuscular time delay (*t_d_*) for most participants fell within the range of 0.1 to 0.2 s, which is consistent with the physiologically plausible delay of 0.1–0.2 s reported in previous studies [17].

To verify the reproducibility of each parameter identification, the intraclass correlation coefficient ICC (1, 1) was assessed for the identified parameters using a two-day test. The results were 0.57, 0.65, 0.67, and 0.73 for *K_p_*, *K_d_*, *K_h_*, and *t_d_*, respectively. These findings suggest that the reproducibility of the identified parameters was moderate in this study.

### 3.4. Trade-Off Between Two Control Targets

As established in the model formulation, COM control and head acceleration control are conflicting objectives during support surface sway. To investigate whether there are individual differences in prioritizing these two control targets, the correlation between the head acceleration feedback gain (*K_h_*) and the maximum real part of the characteristic roots of the identified system (*σ*_max_) was examined. Figure 7 presents a scatter plot of all individual results across both testing days to illustrate this relationship further. On day 1, the correlation coefficient was *r* = 0.87 (*p* < 0.001), and on day 2, it was *r* = 0.93 (*p* < 0.001). These results indicated that an increased emphasis on head acceleration control is associated with diminished stability of upper-body COM control. Regarding the stability of the identified system, the maximum real part of *σ*_max_ was negative for most participants, confirming a mathematically stable system. However, as shown in Figure 7, a few participants exhibited marginally positive roots. These individuals prioritized head acceleration control.

### 3.5. Sensitivity Analysis and Parameter Identifiability

The local sensitivity analysis showed a similar sensitivity pattern across the two measurement days. Based on the root-mean-square (RMS) sensitivity, *K_d_* exhibited the highest sensitivity, followed by *K_p_*, *t_d_*, and *K_h_*, with corresponding RMS values of 1.72, 1.57, 1.34, and 0.79 on Day 1, and 1.63, 1.54, 1.22, and 0.74 on Day 2, respectively. The peak sensitivity of *K_p_* occurred at low frequencies (0.30–0.35 Hz), whereas *K_d_* reached its maximum sensitivity at 0.60–0.70 Hz. In contrast, *K_h_* and *t_d_* exhibited their highest sensitivities at the upper bound of the analyzed range (1.50 Hz) on both days.

The singular values and right singular vectors of the normalized sensitivity matrix are summarized in Table 2. Both exhibited a highly consistent structure across the two measurement days, with *K_p_* and *t_d_* providing the dominant contributions to the first and third right singular vectors, *K_d_* to the second, and *K_h_* to the fourth. These results indicate that the identified parameters occupied distinct directions in the parameter space, with minimal overlap among the dominant parameter contributions. The corresponding left singular vectors, shown as a function of excitation frequency in Figure 8, exhibited closely matching real and imaginary parts across all four modes between Day 1 and Day 2. The Jacobian rank was four for both days, indicating that all four model parameters were practically identifiable, with corresponding condition numbers of 15.57 for Day 1 and 14.93 for Day 2.

## 4. Discussion

In this study, we characterized seated postural control using a parametric model whose objectives are to stabilize the COM and suppress head acceleration. A key measurement variable in this approach is the displacement of the upper-body COM. While our previous study [11] demonstrated the high estimation accuracy of utilizing dual force platforms and support-surface acceleration measurements in the time domain, the present study successfully extended this approach to frequency-domain analysis. By combining the estimated COM displacement with the COM acceleration for frequencies above 0.65 Hz, we confirmed that the frequency response functions (FRFs) closely matched those derived from optical motion capture measurements, yielding minimal identification errors for the balance control system. This measurement system requires less equipment and space than conventional methods, making it a highly practical method for seated balance assessment.

In the identification of seated balance control, a frequency range of 0.3–1.5 Hz was analyzed. The upper frequency limit was extended to 1.5 Hz, compared with the 0.7 Hz used in the standing posture [22], which was expected to improve the accuracy of the time-delay estimation. Furthermore, as the analyzed frequency range widened and the support surface sway increased, it became necessary to include head acceleration feedback in the model [40]. When identification was performed assuming a system without head acceleration feedback, the estimated time delay fell below 100 ms, which is shorter than the physiologically plausible range reported in previous studies [17], and the model failed to achieve a good fit across the analyzed frequency range.

The proposed method demonstrated moderate reproducibility of the identified control parameters (ICC (1, 1) = 0.57–0.73). Compared with the standing posture [22], the expansion of the frequency range improved the reproducibility of time delay identification from 0.56 in the standing posture to 0.73 in the sitting posture. This suggests that the seated posture may be more suitable for identifying time delays. Furthermore, the ICC for the head acceleration feedback gain *K_h_* was 0.67, indicating moderate reliability. In contrast, the ICCs for the proportional gain *K_p_* and derivative gain *K_d_* of COM control were 0.57 and 0.65, respectively, showing decreases compared with the standing-balance model. From a reliability perspective, these findings suggest that the advantage of seated balance control lies in identifying the time delay and head acceleration feedback gain.

The correlation between the maximum real part of the characteristic roots for COM control (*σ*_max_) and the head acceleration feedback gain (*K_h_*) was strongly positive (*r* = 0.87 on day 1 and *r* = 0.93 on day 2). This relationship reflects the trade-off between COM control and head acceleration control within the assumed model, rather than the participants’ control strategies. Accordingly, larger *K_h_* values reduced the stability margin of the identified COM control system. Because the present analysis identifies a time-invariant linear model from steady-state responses, an identified model may be unstable even when participants maintain a stable seated posture. The model becomes unstable when *K_h_* > 1 [35]. Therefore, this study constrained the maximum value of *K_h_* to 1. If strictly stable systems are required, a stricter upper bound (e.g., 0.95) can be applied. Conversely, if estimating *K_h_* without a stability constraint is prioritized, the upper bound can be removed.

The sensitivity and identifiability analyses demonstrated that all four parameters contributed distinguishable local effects to the frequency-domain response, as indicated by the full-rank sensitivity Jacobian for both measurement days. The sensitivity distribution was consistent with the functional roles of the model parameters. The greater influence of *K_p_* at lower frequencies (0.30–0.35 Hz) is consistent with its contribution to the low-frequency component of the model response, whereas the higher sensitivity of *K_d_* at intermediate frequencies (0.60–0.70 Hz) reflects its influence through the velocity feedback component [41]. The highest sensitivity of *K_h_* and *t_d_* at the upper bound of the analyzed range (1.50 Hz) indicates their increasing contribution toward higher-frequency response components associated with head acceleration feedback and delayed control dynamics. Although *K_h_* exhibited lower sensitivity than the other parameters, it remained practically identifiable, suggesting that its contribution could be distinguished without substantial parameter redundancy. These findings support the practical identifiability of the proposed postural control model under the experimental conditions used in this study.

This study had three main limitations. First, the participants were restricted to young healthy males, meaning that the performance of the identification approach may differ in other populations, such as older adults and individuals with vestibular disorders. Moreover, because this homogeneous sample reduced between-subject variability, it likely represented a conservative condition for the ICC-based reproducibility assessment. Second, nonlinearities related to the sway amplitude or frequency were not considered in this study. Different perturbation conditions may lead to discrepancies between the experimental data and the model or alter the balance between COM control and head acceleration control. This study focused on the support surface sway with a peak acceleration of 0.3 m/s^2^ over a frequency range of 0.05–1.5 Hz. Third, this study did not directly verify whether the identified head acceleration feedback gain quantitatively reflects vestibular sensitivity. Although we consider this interpretation reasonable within the proposed control law, direct validation requires further experiments.

Because the single-degree-of-freedom assumption holds up to approximately 2 Hz in the seated posture, this method allows the use of higher perturbation frequencies than in the standing posture. This higher frequency range necessitated the incorporation of head acceleration feedback into the model. Under the eyes-closed condition, identifying this feedback term is theoretically equivalent to an indirect evaluation of vestibular detection capability; however, this interpretation has not been directly verified in this study and remains a topic for future studies. Finally, the practical measurement system and seated test configuration may allow the evaluation of time delay and head acceleration feedback in a wide range of participants.

## 5. Conclusions

This study characterized seated postural control using a parametric model and force platform-based identification approach under horizontal perturbations. The proposed method evaluated concurrent control objectives, including stabilizing the upper-body COM and suppressing head acceleration, with less equipment and space than conventional motion-capture systems. The identified control parameters exhibited moderate reproducibility across testing days. Furthermore, the method captured individual differences in postural control strategies, specifically the trade-off between COM stability and head acceleration feedback. By enabling analysis over a higher frequency range than the standing posture, the seated configuration provides a practical means of evaluating the individual time delay and head acceleration feedback. Because the assessment is performed in a seated position and does not require a standing task, it may also support the application of the method to a wide range of participants.

## Figures and Tables

**Figure 1:**
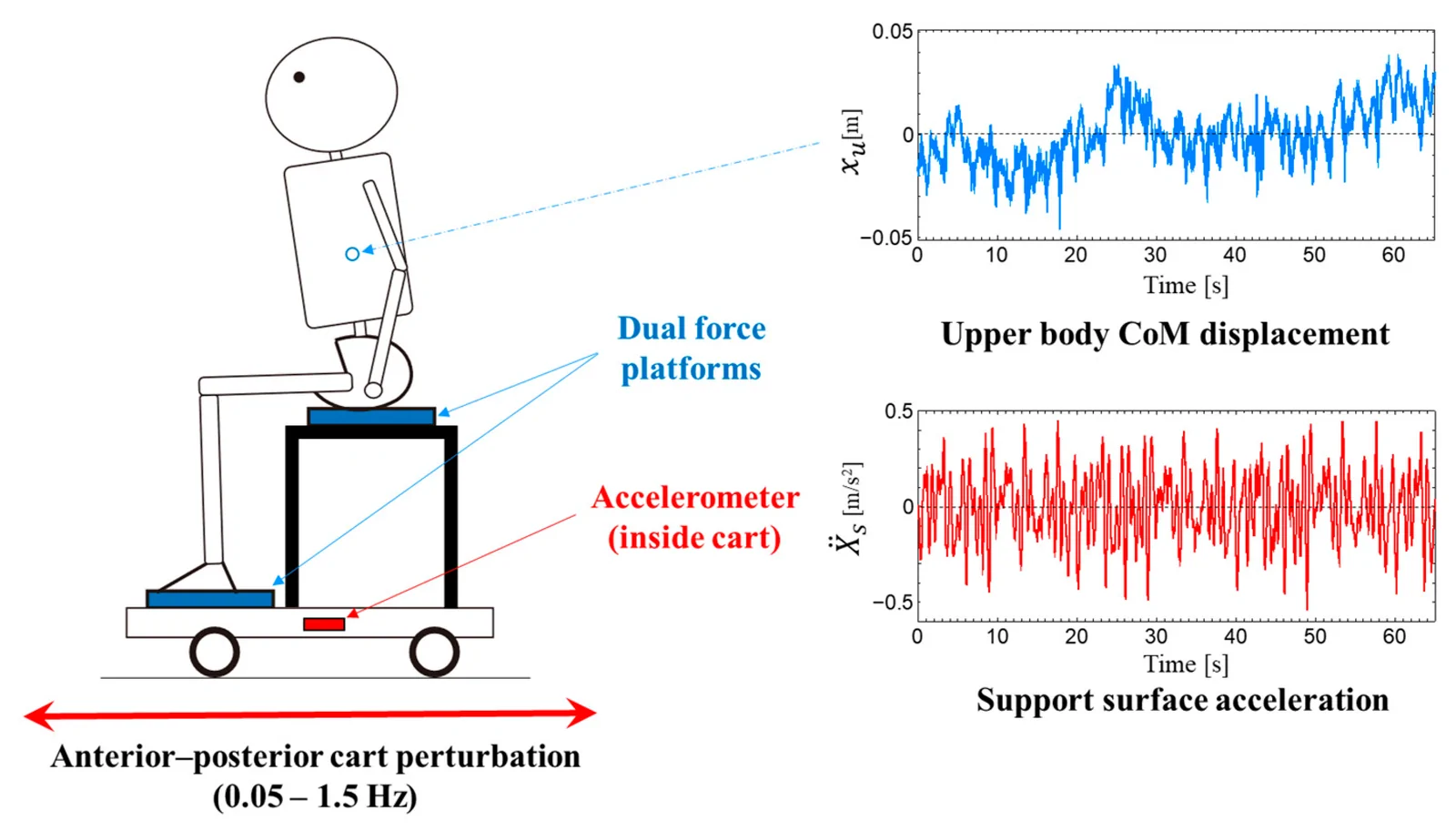
Experimental setup for seated postural perturbation. The motorized cart generated unpredictable anteroposterior (AP) translational perturbations (forward and backward) to evaluate the postural control.

**Figure 2:**
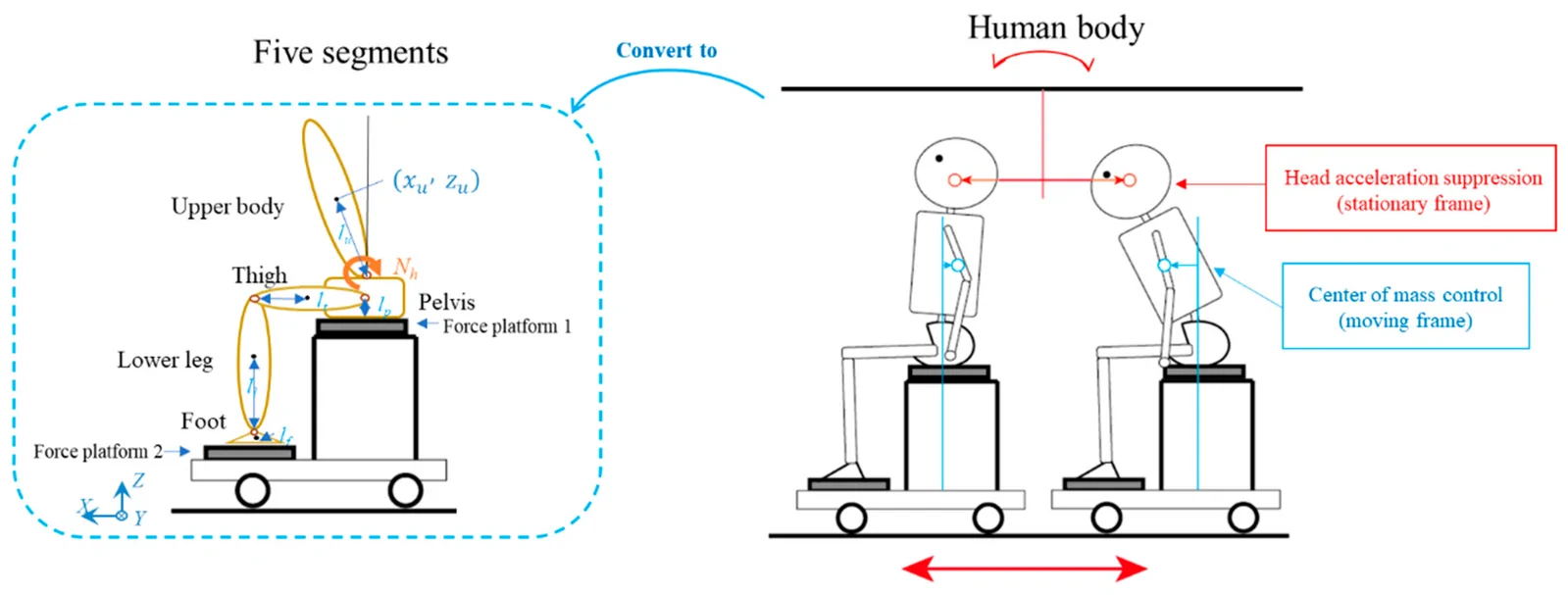
Schematic diagram of the seated balance model and coordinate systems showing COM motion in the moving coordinate system (*p*–*xyz*) and head acceleration in the stationary coordinate system (*O–XYZ*). The red double-headed arrow indicates the direction of cart motion.

**Figure 3:**
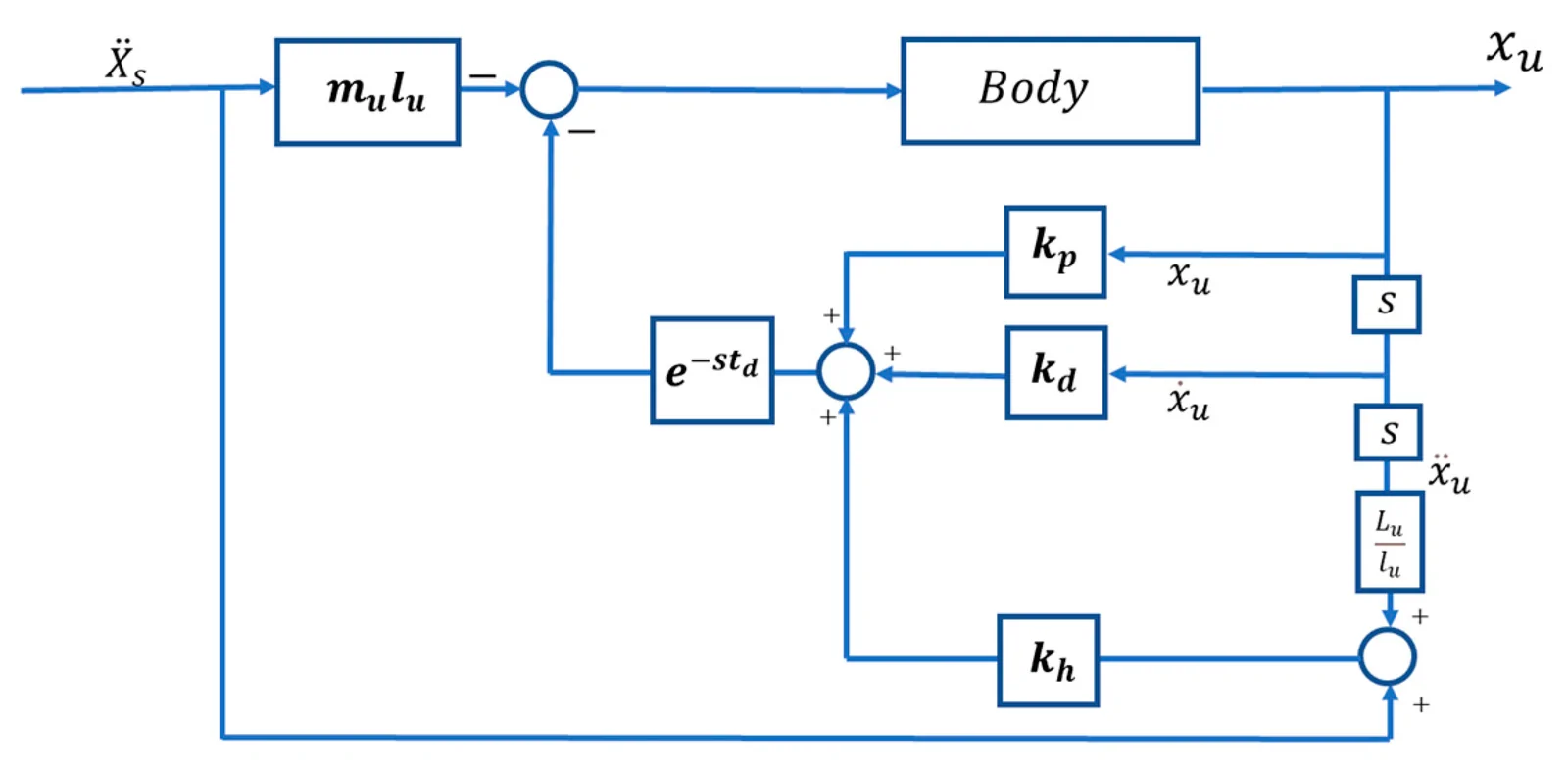
Block diagram of the control system model used to represent the seated postural control.

**Figure 4:**
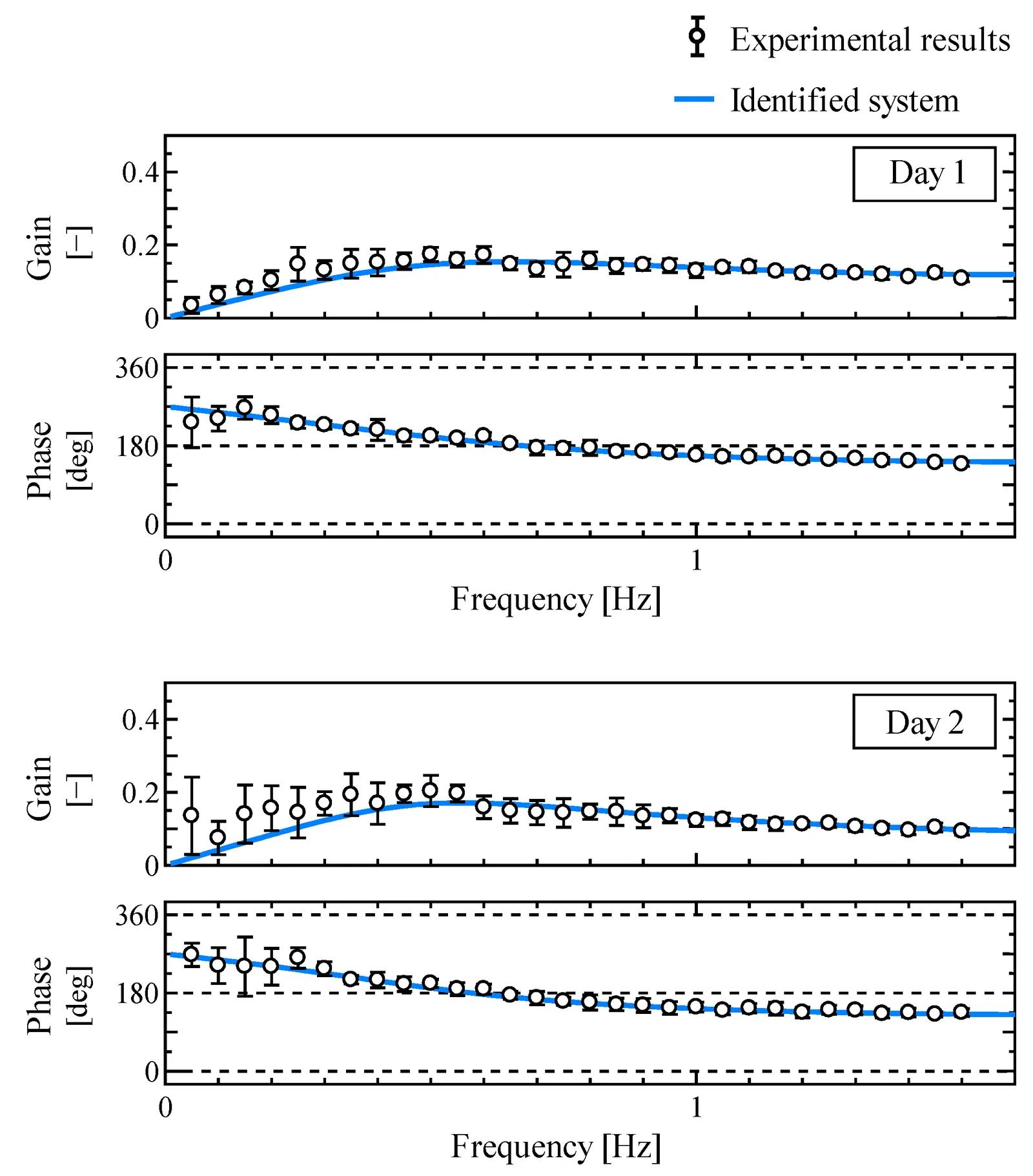
Frequency response diagram of a representative participant comparing days 1 and 2.

**Figure 5:**
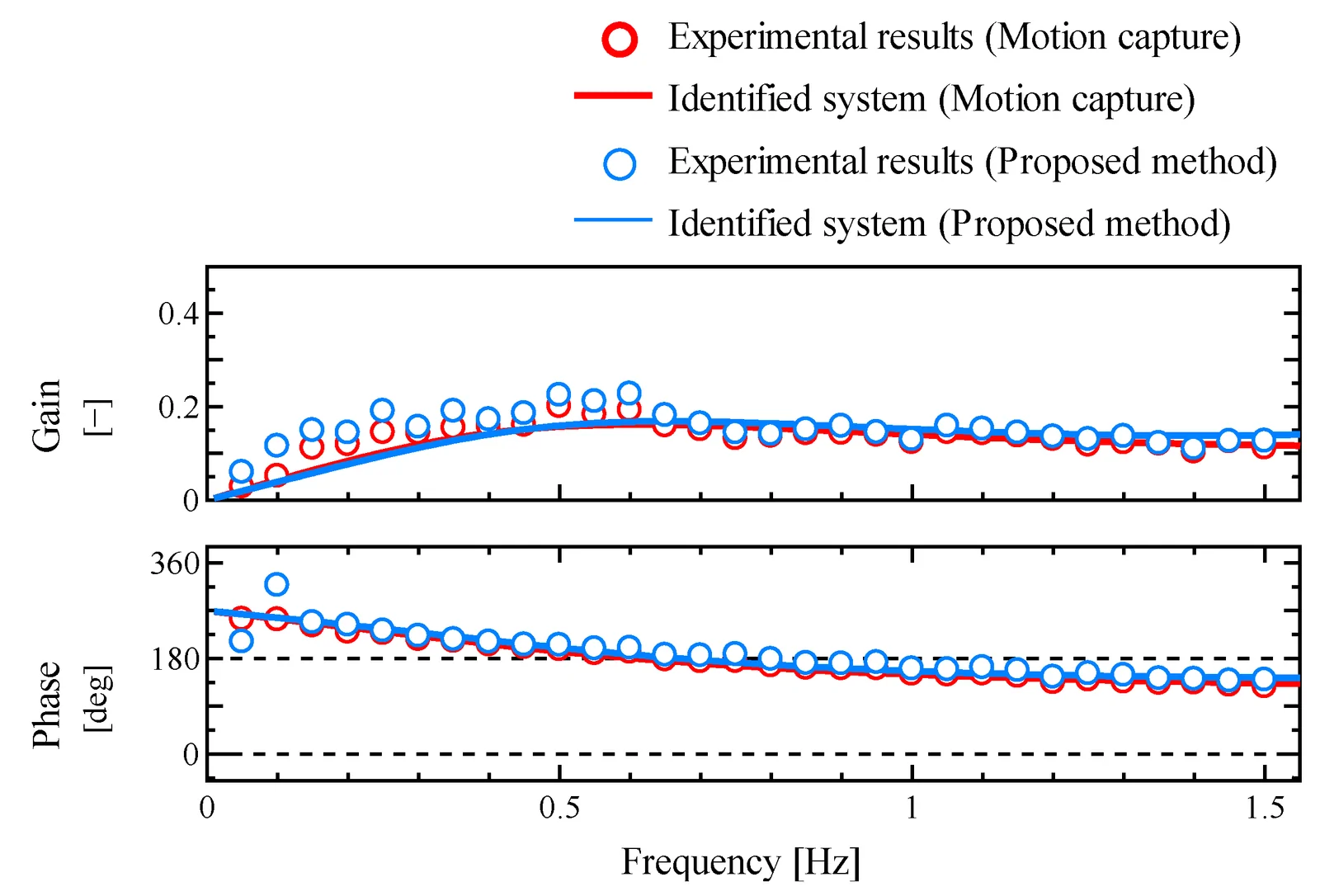
Frequency response diagram of COM estimation derived from the proposed method and optical motion capture system for a representative participant.

**Figure 6:**
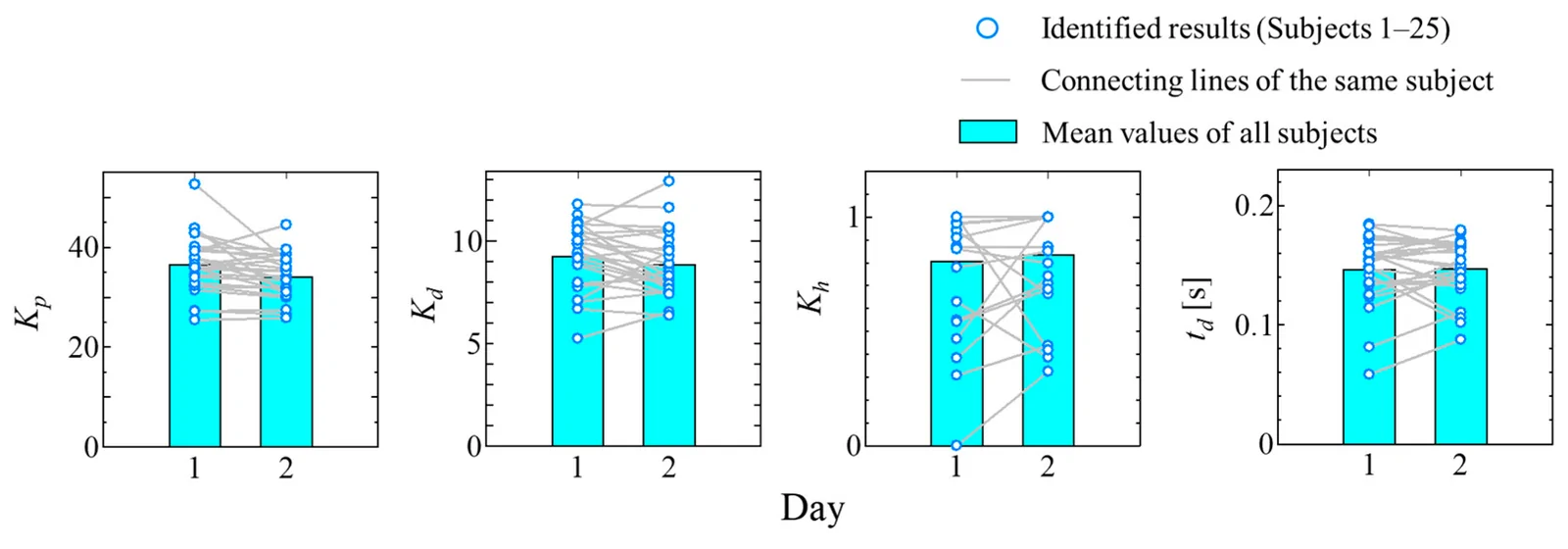
Group-averaged and individual subject values of identified control parameters (*K_p_*, *K_d_*, *K_h_*, and *t_d_*) from the two-day experiment. The bars indicate the group means, and the circles represent the individual subject results.

**Figure 7:**
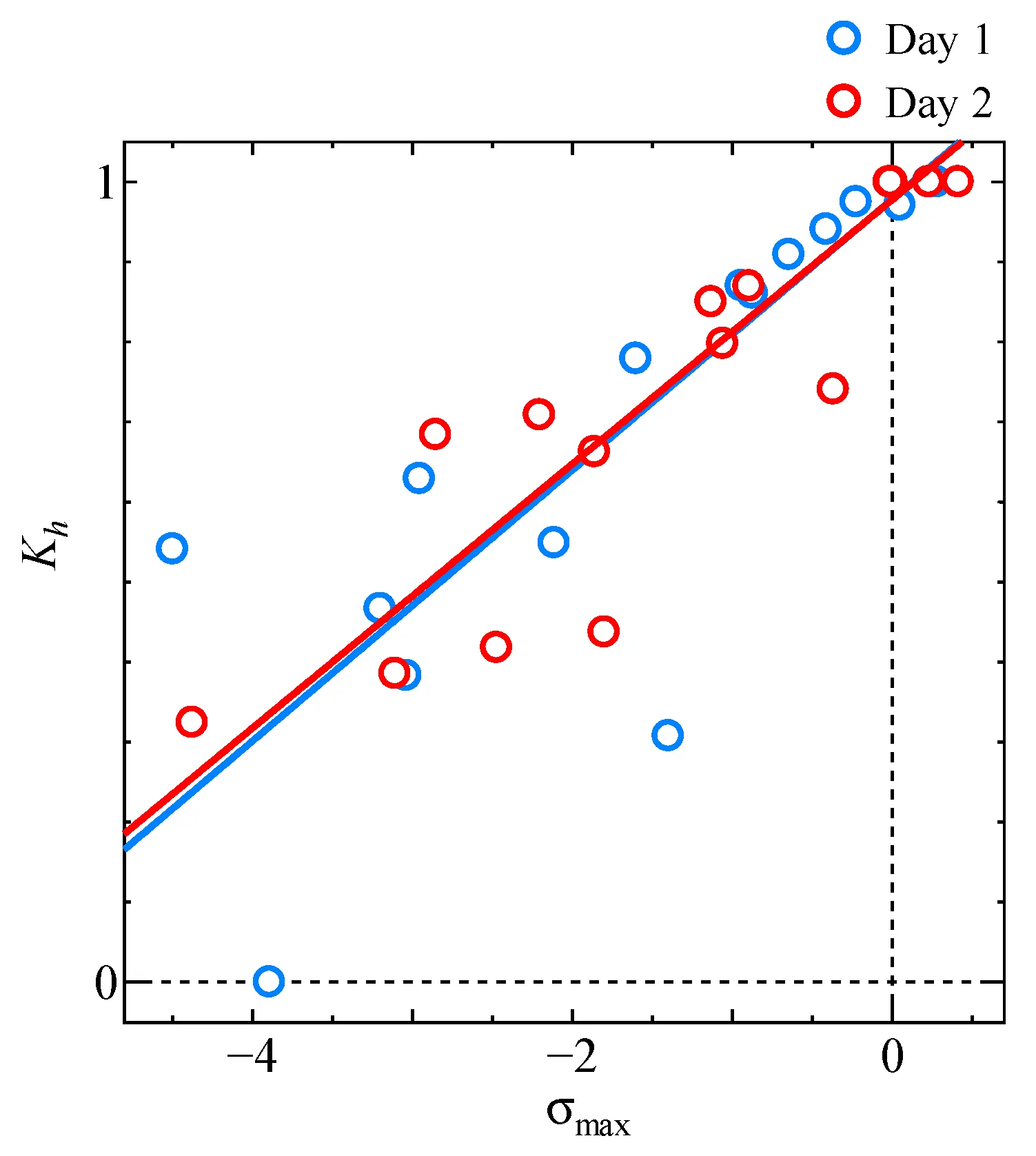
Correlation between normalized head acceleration feedback gain (*K_h_*) and maximum real part of characteristic roots (*σ*_max_) during seated balance control. Blue and red lines indicate the linear regression fits for Day 1 and Day 2, respectively. Note: Some *K_h_* points reached the model’s upper limit, causing visual overlap; all participants’ data were included in the analysis.

**Figure 8:**
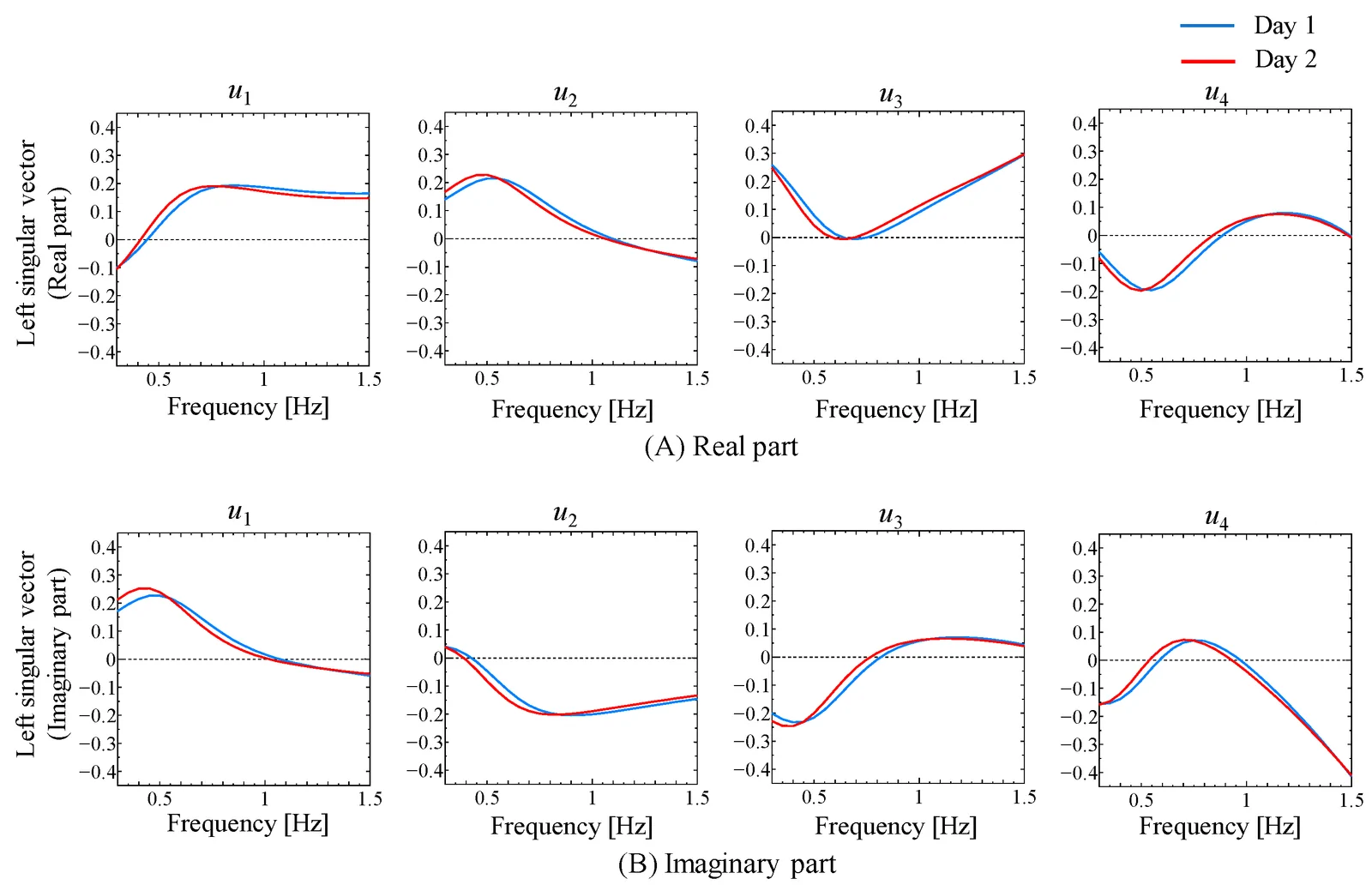
Real and imaginary parts of the left singular vectors (*u*_1_–*u*_4_) of the normalized sensitivity matrix as a function of excitation frequency for Day 1 and Day 2.

**Table 1:** Physical parameters of each body segment as functions of total body mass *M* [kg] and height *H* [m]. While all participants in this study were male, parameters for females are listed for reference.

**Segment**	**Symbol**	**Male**	**Female**
Upper body	*m_u_*	0.534*M*	0.504*M*
	*J_u_*	0.0099*MH*^2^	0.0105*MH*^2^
	*l* *_u_*	0.173*H*	0.184*H*
	*L_u_*	0.470*H*	0.470*H*
Pelvis	*m_p_*	0.122*M*	0.122*M*
	*l_p_*	0.025*H*	0.022*H*
	*L_p_*	0.050*H*	0.044*H*
Thigh	*m_t_*	0.220*M*	0.246*M*
	*l_t_*	0.102*H*	0.107*H*
	*L_t_*	0.195*H*	0.198*H*
Lower leg	*m_l_*	0.102*M*	0.106*M*
	*l_l_*	0.146*H*	0.143*H*
	*L_l_*	0.246*H*	0.243*H*
Foot	*m_f_*	0.022*M*	0.022*M*
	*l_f_*	0.016*H*	0.016*H*
	*L_f_*	0.039*H*	0.039*H*

**Table 2:** Singular values (*σ*) and right singular vectors (*V*) of the normalized sensitivity matrix for Day 1 and Day 2. (**A**) Singular values; (**B**) Right singular vectors.

(**A**)								
**Component**			**Day 1**			**Day 2**		
*σ*_1_			10.12			9.55		
*σ*_2_			8.86			8.40		
*σ*_3_			3.85			3.72		
*σ*_4_			0.650			0.639		
Condition number			15.6			14.9		
(**B**)								
**Parameter**	***v*** **_1_**		***v*** **_2_**		***v*** **_3_**		***v*** **_4_**	
	**Day 1**	**Day 2**	**Day 1**	**Day 2**	**Day 1**	**Day 2**	**Day 1**	**Day 2**
*K_p_*	0.708	0.747	−0.221	−0.210	−0.654	−0.615	−0.152	−0.141
*K_d_*	−0.205	−0.187	−0.941	−0.942	0.148	0.149	−0.225	−0.237
*K_h_*	−0.307	−0.302	0.248	0.244	−0.208	−0.246	−0.895	−0.888
*t_d_*	0.602	0.562	0.065	0.097	0.712	0.734	−0.355	−0.368

## Data Availability

The datasets generated and analyzed during the current study, including the measured values of all experiments, frequency response functions, and identification results for all subjects, are available in the figshare repository, https://doi.org/10.6084/m9.figshare.32598009.
